# Migration and Malaria in Europe

**DOI:** 10.4084/MJHID.2012.014

**Published:** 2012-03-10

**Authors:** Begoña Monge-Maillo, Rogelio López-Vélez

**Affiliations:** Tropical Medicine. Infectious Diseases Department. Hospital Ramón y Cajal, Madrid, Spain

## Abstract

The proportion of imported malaria cases due to immigrants in Europe has increased during the lasts decades, with higher rates associated with settled immigrants who travel to visit friends and relatives (VFRs) in their country of origin. Cases are mainly due to *P. falciparum* and Sub-Saharan Africa is the most common origin. Clinically, malaria in immigrants is characterised by a mild clinical presentation including asymptomatic or delayed malaria cases and low parasitic levels. These characteristics may be explained by a semi-immunity acquired after long periods of time exposed to stable malaria transmission. Malaria cases among immigrants, even asymptomatic patients with sub-microscopic parasitemia, could increase the risk of transmission and cause the reintroduction of malaria in certain areas that have adequate vectors and climate conditions. Moreover, imported malaria cases in immigrants can also play an important role in the non-vector transmission out of endemic areas, through blood transfusions, organ transplantation or congenital transmission or occupational exposures. Consequently, outside of endemic areas, malaria screening should be carried out among recently arrived immigrants coming from malaria endemic countries. The aim of screening is to reduce the risk of clinical malaria in the individual as well as to prevent autochthonous transmission of malaria in areas where it has been eradicated.

## Introduction

Malaria continues to present one of the major challenges to global public health with an estimated 225 million clinical cases and more than 700000 deaths in 2009, mostly in children under 5 years old from sub-Saharan Africa.^1^ In Europe, malaria is eradicated in almost all countries of the World Health Organization European Region except for Azerbaijan, Georgia, Kyrgyzstan, Tajikistan and Turkey.^1^

In 2010 there were 47.3 million foreign-born people in the European Union (EU), corresponding to 9.4% of the total population. The majority of them, 31.4 million, were born in non-EU countries, while 16 million were born in another EU Member State. Data about those coming from endemic malarial countries is scarce. Estimates indicate that more than 5 million African immigrants could be living in Europe. Among them, about two thirds are from North Africa (Algeria, Morocco and Tunisia) and the rest are from Sub-Saharan Africa (SSA), mostly from West Africa (Ghana, Nigeria and Senegal). About 4 million come from South East Asia and nearly 2.2 million from Latin America.^2^

**Fig 1:**
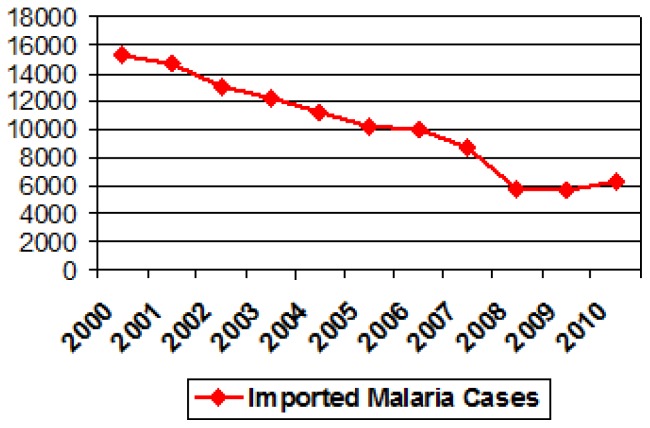
Imported malaria cases in Europe. Data from WHO Regional Office for Europe.

Imported malaria is defined as an infection acquired in a malaria endemic area but diagnosed in a non-endemic country. The malaria Programme of the WHO European Region annually collects data of malaria cases from 51 countries in the region. It reported an eight-fold increase in the number of imported malaria cases between 1972 and 1988 (from 1500 to 12000 cases), followed by a more gradual rise in 2000 (15500 cases). Most of the cases were imported to Western Europe, with France, the United Kingdom (UK), Germany and Italy accounting for more than 70% of all cases.^3^ Although, in the last decade data from the World Health Organization (Figure 1) has shown a progressive decrease in the global incidence of imported malaria in most European countries,^4^ despite a slight rise in cases reported from 2008.^5^ Several published studies have corroborated such decreases in certain European countries such as the Netherlands^6^ and the UK^7^ which could be explained by a reduction in the global malaria transmission in SSA countries.

Today, the profile of immigrants is changing with higher rates of immigrants from southern (malaria-endemic) areas moving to northern (malaria free) industrialised areas of the world.^3^ The proportion of imported malaria cases due to immigrants in Europe has increased during the last few years from 14% in research published more than 10 years ago, to 86% in more recent studies.^8^ On pooling the reports, nearly 43% of malaria cases registered in key European centres occurred in non-nationals. The rates of malaria are much higher in settled immigrants who travel to visit friends and relatives (VFRs) in their country of origin. They can account for up to 70% of the cases in several reports.^9^,^10^

## Methods

A literature review was conducted using MEDLINE, EMBASE, Web of Science and the Cochrane Library database. The review included case studies, reviews and expert opinion. Search was based on published articles on imported infectious diseases in general and specifically malaria. Only those including data relating to immigrants (adult and paediatric) and based in European countries were included. A table with the most relevant characteristics of imported malaria among immigrants in Europe was created. Data referring to visiting friends and relatives (VFRs) and to refugees was not included. The selection criterion for including articles was based on the subjective opinion of the authors who considered article relevancy to the current research.

## Imported malaria in immigrants and in immigrant-travellers (VFRs)

Most of the imported malaria cases among immigrants in Europe are due to *P. falciparum* and SSA, in particular West Africa, is the most common origin.^11^–^15^ The incidence of infection by *P. ovale* and mixed infections is very similar to the incidence found in West Africa, which is probably due to the very high number of immigrants from these areas. Data from several of the selected published reports about imported malaria among immigrants in different European countries is summarised in Table 1.

Children account for around 15–20% of all imported malaria cases in Europe and are increasing, as more children travel. Similarly as in adults, *P. falciparum* is the most frequently identified species and SSA is the most common origin.^8^–^16^ When comparing clinical malaria among adults and children, the latter are less likely to complain of chills, arthralgia/myalgia or headaches. Instead they are more likely to present with non-specific symptoms or with gastrointestinal symptoms. They also have hepatomegaly and jaundice more often than adults.^16^ In general, children seem to have a higher risk of severe malaria than adults worldwide. For imported cases, the risk factors for developing severe malaria include young age (< 5 years), delayed diagnosis (probably due to cultural and language barriers that make it difficult to access health care systems) and non-immunity to malaria.

There is a specific group of immigrants who, once settled in the host country, travel to their countries of origin to visit friends and relatives (VFRs). They have been described as a special risk group for certain travel-related illnesses, especially infectious diseases, when compared to other types of travellers. This is because they usually travel to high-risk destinations, for longer periods of time and usually do not seek pre-travel advice.^17^–^19^ This is especially significant in the case of malaria, where returned VFRs make up the largest proportion of malaria cases reported in many developed countries^15^ mainly due to *P. falciparum (*75.8%) and almost exclusively in patients from SSA.^12^,^20^,^21^ In a Spanish report, they accounted for 37% of malaria cases notified during the study period.^12^ Among UK surveillance data of imported malaria from 1978 to 2006, 64.5% of the cases were VFRs 21. The GeoSentinell report published in 2006 found that immigrant VFRs who travelled to SSA had more than eight times the odds of receiving a diagnosis of malaria than a tourist who visited this region. Immigrant VFRs also had more than twice the odds of receiving a diagnosis of malaria after travel to Asia and more than three times the odds after travel to Latin America when compared with tourist travellers.^20^

The higher risk VFRs have of acquiring malaria is due to diverse factors. First of all they travel to high malaria endemic areas. Among the different reports published about VFRs, the most common origin was SSA.^12^, ^20^, ^21^ Moreover, they usually travel during the Northern Hemisphere’s summer months, which coincide with the rainy season in West Africa and with monsoon season in the Indian subcontinent. VFRs are also more likely to be travelling to rural areas and destinations not developed for tourism, often with a poor health infrastructure. They also usually stay at their destination for long periods of time.^22^

VFRs commonly believe they are immune to malaria,^17^ but such immunity seems to disappear some years after arriving in Europe. This makes them as vulnerable as other travellers to developing symptomatic and even severe malaria when travelling “home”. Moreover, VFRs are more likely to travel with small children or while pregnant when compared with other types of travellers. In both these cases, VFRs are groups with a higher risk of contracting severe malaria. The low perception of risk makes them rarely seek pre-travel advice. When advice is sought, it is more likely to be from a general practitioner who may not be up to date on pre-travel recommendations. This means that anti-malarial prophylaxis is used less frequently or taken incorrectly.^22^ Cost may also be an impediment to taking antimalarials. This may lead people to purchase the drugs at their destination where the quality of the drug may be substandard. In other cases no pre-travel advice is sought because travel is undertaken at the last minute in order to attend family events such as marriages, funerals or to visit sick relatives. Sometimes language and cultural barriers may prevent VFRs from seeking medical advice.^23^

## Semi-Immunity to Malaria Among Immigrants

Malaria in immigrants is characterised by milder clinical presentation, lower parasitic levels, shorter time for parasite clearance after treatment and shorter fever duration than malaria in travellers.^15^,^24^ Even asymptomatic malaria cases among recently arrived immigrants coming from African malaria endemic countries have been described. In a Spanish series on imported infectious diseases by immigrants, among SSAs 7.1% of malaria cases due to *P. falciparum* were asymptomatic at the moment of diagnoses.^25^ A study conducted in Italy to determine the prevalence of malaria among asymptomatic SSA immigrants assessed by nucleic acid sequence based amplification found a 31.8% prevalence of malaria.^26^

Much more is known about the prevalence of malaria and about the number of asymptomatic cases among refugees. This is probably due to established protocols on screening for infectious diseases that are required of refugees to enter certain host countries. The reported prevalence of asymptomatic malaria in SSA refugees screened post arrival ranges from 2.4% to 31.8%.^27^–^29^ Although studies have evidence showing the role of asymptomatic *Plasmodium spp* infection in populations resident in Latin America, less is known about imported asymptomatic cases among immigrants or refugees from the region.^30^, ^31^

Imported malaria cases among immigrant children coming from endemic malaria countries can also be linked to milder symptoms than those in child travellers born in non-endemic areas.^15^ Even evidence of asymptomatic cases among immigrant children has been published. Most reported data for newly arrived SSA refugee children who present with malaria shows a prevalence of 6% to 32% with high rates of asymptomatic cases.^32^, ^33^, ^34^

Another characteristic of imported malaria among immigrants are the cases of late clinical presentation of *P. falciparum* infections. Most *P. falciparum* infections occurred in the first three months after arrival, with the delayed onset of clinical malaria characteristics of other *Plasmodium* species. Cases of prolonged *P. falciparum* malaria have been described two,^35^ four,^36^ or even eight years^37^ after living outside of an endemic area. In all of these cases other risk factors such as travel to other malaria endemic areas, blood transfusions, visits to an airport or contact with a person suffering from malaria had been excluded. A case control study performed in France tried to identify the incidence and the factors associated with prolonged *P. falciparum* infection out of endemic area. They analysed 2680 patients who had travelled or lived in malaria endemic regions and that were diagnosed with a *P. falciparum* infection. Case patients had an infection diagnosed > 59 days after arrival in France, and control patients had infections detected ≤30 days after their arrival. Late infections were detected in 2.3% of the patients with a median diagnoses delay of 5 months (interquartile range 3–9 months). Three independent factors were positively associated with prolonged *P. falciparum* infections, these were: being a first arrival immigrant, being a pregnant woman, and taking mefloquine prophylaxis.^38^ Undoubtedly many more studies are needed to determine which factors can condition a late presentation of clinical malaria. Other cases of late *P. falciparum* malaria infections have been published, but these studies found other risk factors, such as the recent arrival of a member of the family from a malaria endemic area who could have transported an infected *Anopheles* in their luggage.^39^, ^40^

Asymptomatic malaria cases or clinical symptoms long after arrival may be explained by a semi-immunity status in the migrant. This semi-immunity seems to be related to prolonged and intense exposure and is usually acquired in areas with persistent malaria transmission as well as in areas of seasonal transmission.^41^,^42^ Immunity to malaria infection is relatively slow to develop and incomplete, although immunity to death is acquired more quickly and may be important after a single episode. This semi-immunity does not avoid the infection and the establishment of peripheral blood parasitemia, but seems to protect against several malaria infections. So, in general, people from malaria endemic countries may have sub microscopic levels of parasitemia, but not feel ill or may present atypical features of malaria.^43^

The mean time semi-immunity persists after leaving the transmission area has not yet been clearly established. Malaria immunity has been reported to rapidly wane after the end of expose to the parasite, which suggests that continued exposure to malaria antigens seems to be required. Nor is there agreement on what components of immunity to malaria are lost without exposure, generally this loss in identified only by the fact that such people do experience symptomatic infections.^44^ However, a study conducted in France gives evidence that malaria among African adult immigrants is less severe (lower parasite density and lower frequency of severe and complicated diseases) and more readily cured than in Europeans, even more than 14 years after living out of an endemic area.^45^ An in vitro study also showed that humoral and cellular responses to defined *P. falciparum* antigens persisted in migrants from West Africa who spent up to 13 years in France without travelling to their country of origin.^46^ This is consistent with the results of two observational studies in the highlands of Madagascar (which is a non-malaria endemic area) during the 1987 malaria outbreak. Patients who were more than 40 years old, who had spent their childhood in malaria hyperendemic areas before control programmes for malaria existed, were found to be more protected against clinical *P. falciparum* infection than younger patients. They also had a stronger humoral and cellular immune response to *P. falciparum* antigens.^47^ The persistence of the semi-immunity is especially relevant in VFRs. If such immunity is lost, immigrants who travel to their country of origin would have the same risk as travellers born in non-endemic countries of suffering severe malaria.

## Migration and Risk of Induced and Reintroduced Malaria

Migration could increase the risk of transmission and reintroduction of malaria in certain areas where it has previously been eradicated. Adequate climate conditions and the presence of malaria vectors in certain countries could help create a local vectorial transmission and a reintroduction of malaria.^48^,^49^ Moreover, asymptomatic patients with sub-microscopic parasitemia are also capable of infecting mosquitoes, and are thus considerate as reservoirs of malaria.^50^

Malaria cases imported by immigrants can play an important role in the non-vectorial transmission out of endemic areas through other mechanisms of transmission such as blood transfusions, organ transplantation, congenital transmission or occupational exposure.^51^,^52^

Data on the frequency of transfusion transmitted malaria in non-endemic countries shows a rate of about 0.2% and the most frequently associated species are *P. falciparum, P. malariae* and *P. vivax*.^53^,^54^ In most of the published cases the donor was foreign born and came from an endemic area. Among five cases reported in the UK, four of the donors came from a SSA country; the fifth case was a UK traveller to Africa. In all cases *P. falciparum* was the isolated species.^53^ Another interesting case of transfusion transmitted *P. falciparum* malaria occurred in France: the donor also came from SSA with the peculiarity that he had been living in France for four years with no history of fever or illness.^51^

Malaria is an unusual complication of solid-organ transplantation in non-endemic countries. Most cases have been described after renal transplantation^52^,^55^,^56^ with a few cases reported after heart^57^ or liver transplantations.^52^,^58^,^59^ Any species of *Plasmodium* may be involved.^60^ Although there are cases where the donor was a traveller to an endemic area,^58^ in most of the published cases donors are immigrants coming from a country with a high prevalence of malaria. In one case of *P. falciparum* transmission through a heart transplant in France, the donor came from SSA and had been asymptomatic during the 15 months she lived in the host country.^57^ In a published case of a multi-organ donation in Germany *P. vivax* was transmitted to receptors: the donor was an immigrant coming from SSA who had lived in Germany for the last 18 months and had been asymptomatic during all of that time.^61^

Congenital malaria out of endemic areas is rare and predominantly seen in infants of recently arrived mothers. Most infants develop symptoms at the age of approximately one month. Because the initial clinical presentation of congenital malaria can be very similar to that of neonatal sepsis, physicians must be aware and suspect *Plasmodium* infection among those born to immigrant mothers.^62^

In Europe, where malaria has been eradicated since 1975, *Anopheles* spp. mosquito vectors remain prevalent in parts of Southern and Central Europe. In fact, transmission of malaria to local residents has been reported over the last 10 years, and the hypothesis of malaria reintroduction has also recently been confirmed. In Greece, in 2011, at least 33 autochthonous malaria cases due *to P. vivax* were recorded.^63^,^64^ In Spain another case of autochthonous *P. vivax* infection was detected.^65^ We must also consider global climate change, which could help spread malaria to northern latitudes.^66^ However, the real possibility of a European country becoming an endemic malaria area seems quite small. Surveillance and public health measures would probably avoid such situation.

## Screening for Malaria Among Immigrants

The evidence of asymptomatic malarial infections or the late clinical presentation of these infections, which can be reservoir of malaria added to other possible mechanisms of transmission out of endemic area raises the need of systematic screening processes. Screening has been more established for refugees, where approaches to mitigate the risk of malaria in these populations include mass antimalarial treatment pre-departure or on arrival and screen and treat strategies for targeted groups. The US Center for Disease Control (CDC) issued new guidelines in 2010 for refugees arriving from SSA which recommend presumptive treatment either pre-departure or on arrival.^67^ Recent guidelines from the Australian Society for Infectious Disease include malaria testing and treatment at both pre-departure and at the post-arrival health assessment.^68^ These measures should probably be extrapolated to recently arrived immigrants coming from malaria endemic countries. Screening for malaria among this population could constitute an effective public health measure, especially when a high proportion of immigrants can be asymptomatic.^69^

The effectiveness of strategies based on screening at arrival is heavily dependent on the proportion of refugees and immigrants who receive systematic assessment by a clinic with specific expertise in imported infectious diseases and, the diagnostic method used.^27^ In fact, diagnosing asymptomatic patients requires techniques that enable the measurement of infections with a very low density of parasitemia. The sensitivity of a malaria test is the probability that at least one parasitized red blood cell is detected. This is directly related to the volume of blood that the test can screen and the density of parasites in the blood sample.^70^ With microscopy techniques the volume of blood examined is 0,06–0,2 μL, while PCR uses several micro-litres, giving theoretical limits of detection of 5–16 parasites/μL by microscopy and 0.002–1 parasites/μL by PCR.^71^ A systematic review and meta-analysis of surveys of endemic populations in which *P. falciparum* prevalence is measured by both microscopy and PCR techniques indicated that the prevalence of infection measured by microscopy was, on average, 50.8% of that measured by PCR.^72^ A study conducted in the United States comparing a rapid antigen capture enzyme assay with PCR for the screening in SSA refugees showed that the sensitivity and specificity of the rapid antigen assay were 22% and 66% respectively.^73^ In Canada two different antigen detection tests were also evaluated: ICT Malaria (sensitivity 37.5% and specificity 100%) and OptiMAL (sensitivity 29.1% and specificity 95.6%).^27^ In both studies microscopy was also analysed without better outcomes. Therefore, PCR is probably by far the most powerful tool for such surveillance.

Moreover, taking into account the long periods of time after arrival that parasitemias seem to persist, we should consider whether screening should be offered not only to recently arrived immigrants but also immigrants settled in the host country for some time.^69^ In fact, a mathematical modelling has estimated the maximum duration of *P. falciparum* infection after interruption of transmission in about 4 years.^74^

## Summary

Malaria imported by immigrants makes up a significant proportion of the cases diagnosed out of endemic areas. They are mostly due to *P. falciparum* and found among those coming from West Africa. Malaria in immigrants is characterised by a mild clinical presentation, low parasitic levels; short parasite clearance time after treatment and, short fever duration. In fact, a high proportion of immigrants may be asymptomatic or present clinical malaria long after arrival in the host country. Such characteristics seem to be explained by the semi-immunity to malaria acquired after living in endemic areas. In parallel with migration, immigrants settled in the host country increasingly travel to their country of origin to visit friends and relatives (VFRs). They have been described as a special risk group for certain travel related infectious diseases when compared to other types of travellers. Malaria is one of the most frequent diagnoses among them, mainly due to *P. falciparum* and almost exclusively in patients from Sub-Saharan Africa (SSA). Congenital transmission or transmission due to blood transfusion or organ transplantation out of endemic area has been described. Furthermore, adequate climate conditions and the presence of malaria vectors in certain European countries could contribute to local vectorial transmission and a reintroduction of malaria in areas where it had been eradicated. Consequently, out of endemic areas, strategies to control imported malaria by immigrants should be multifaceted. Firstly, screening for malaria among recently arrived immigrants from malaria endemic countries should be performed. The aim of this is to reduce the risk of clinical malaria in the individual as well as to prevent autochthonous transmission of malaria in areas where it has been eradicated. The evidence of persistent asymptomatic parasitemia suggests that screening time after arrival could also be considered. Secondly, the relevance of imported malaria cases among VFRs highlights the need of preventive health promotion strategies culturally adapted and focused on pre-travel advice.

## Figures and Tables

**Table 1:** Data from several published European series of imported malaria by immigrants in different European countries.

Author. Country. Year	Nº of cases	Adults Children	Area of origin	Malaria species	Severe cases
Espinosa-Vega E et al.^75^ Spain, 2011.	20	11 Adults 9 Children	19 SSA (11 Central Africa; 8 West Africa), 1 America	18 *P.falciparum,* 2 *P.vivax*	1
Antinori S et al.^76^ Italy 2011.	35	35 Adults	NS general data from different groups (75.3% Africa, 10.7% Asia, 7.7% Indian Subcontinent, 4.6% South-America, 1.5% Middle East).	NS general data for different groups (78.3% *P.falciparum*, 16.5% *P.vivax*, 3.1% *P.ovale*, 0.3% *P.malariae*, 1.7% mixed infections)	NS general data for different groups 15% criteria for severe malaria.
Gracía-Villarrubia M et al.^77^ Spain 2011.	55	55 Children	NS general data from different groups (83.9% Africa, 8.6 % Asia, 1.7 America)	NS general data for different groups (69.5 *P.falciparum*, 14.4 *P.vivax*, 5.7% *P.ovale*, 4.6 *P malariae*, 2.9% mixed, 2.9% *P.spp*).	0
Arnáez J et al.^78^ Spain, 2010.	46	46 Children	45 SSA, 1 Latin America	41 *P.falciparum,* 5 mixed infections (NS)	0
Rey S et al.^79^ Spain, 2010	19	19 Adults	NS general data for different groups 98.2% SSA, 1.8% Asia.	NS general data for different groups (94.7% *P.falciparum*; 5.3% *P.ovale*)	0
Pistone T et al.^71^ France, 2010			NS general data for different groups 82.9% ASS	NS general data from different groups (82 % *P.falciparum*, 6% *P.ovale*, 8% *P.vivax* and 2% *P.malariae*).	NS
Mascarello M et al.^10^ Italy, 2009	35	35 Adults	SSA: 34 West Africa. 1 other East, Central or Southern African countries	All *P.falciparum*	0
Monge-Maillo et al.^25^ Spain, 2009	212	69 Children 15 Young Adults 128 Adults	199 SSA, 13 Latin America	128 *P.falciparum*, 14 *P.vivax*., 13 *P.malariae*.,10 *P.ovale*., 39 *P.spp*.,8 mixed infections (5 *P.falciparum* & *P.malariae*; 2 *P.falciparum* & *P.ovale*; 1, *P.malariae* & *P.ovale*	NS
Mascarello M et al.^15^ Italy, .2008	50	30 Adults 20 Children	NS general data from different groups ( 94.5% Africa, 4.5% Asia, 1% Oceania and Central and South America)	NS general data from different groups (76.8% *P falciparum*, 9.5% *P.ovale*, 5.3%, *P.vivax* and 2.4% *P.malariae*).	2 (1 adult and 1 children)
Millet JP et al.^12^ Spain, 2008.	106	NS general data from different groups (12% were children)	98 SSA, 4 Asia, 4 Latin America.	NS general data from different groups (81.6*% P.falciparum*)	0
Guedes S et al.^80^ Findland, 2008.	201	145 Children 56 Adults	120 Africa, 19 South East Asia, 72 Unknown	NS general data from different groups (61% *P.falciparum*, 22% *P.vivax*, 10% *P.ovale*, 2%, *P.malariae*).	
Martínez-Baylach J et al.^81^ Spain, 2007.	5	5 Children	SSA	3 *P.falciparum,* 1 *P.vivax,* 1 *P.malariae*	0
Driessen GJ et al.^14^ Netherlands, 2007	8	8 Children	NS genera data from different groups. (84.4% SSA, 15.6% Asia)	NS general data from different groups (81% *P.falciparum*, 3% *P.ovale*, 13% *P.vivax*, 3% *P.vivax* & *P.falciparum*)	
Rojo-Marcos G et al.^13^ Spain, 2007	46	NS general data from different groups ( 42% children)	SSA	NS general data from different groups (89% *P. falciparum*; 7% *P.ovale*; 4% *P. malariae*)	NS
Spinazzola F et al.^82^ Italy, 2006	137	137 Adults	NS general data from different groups ( 50.3% West Africa, 28.4% East Africa, 9.1% South Africa, 8.3% Asia, Central or South America 2.4%,)	NS general data from different groups. (77.5% *P.falciparum*, 18.1% *P. vivax*, 1.4% *P ovale*, 0.4% *P.malariae,* 2% mixed infections )	Overall rate of severe malaria was 11.6% for all groups. Among immigrants 2 patients died 1.5%).
Ladhani S et al.^83^ United Kingdom, 2006	55	55 Children	NS general data from different groups (84% SSA, 15% Indian subcontinent))	NS general data from different groups. (77% *P.falciparum*, 14 % *P. vivax*, 6% *P.ovale*, 3% *P.malariae*)	0
Baas MC et al.^84^ The Netherlands, 2006	26	26 Adults	NS general data from different groups (86% SSA, 7% Asia, 18% Central and South America))	NS data from different groups (82 % *P.falciparum*, 9.3% *P.vivax*, 5% *P.ovale*, 2 % *P.malariae).*	NS
Chalumeau M et al.^85^ France, 2006	7	7 children	SSA	7 *P.falciparum*	NS
Ladhani S et al.^86^ United Kingdom, 2003	38	38 children	34 SSA, 4 Indian subcontinent	NS data from different groups. (91 % *P.falciparum,* 5.7% *P.vivax*, 2.4% *P.ovale*, 0.5 %, *P.falciparum & P.vivax*, *P.falciparum* & *P.ovale.*	NS data from different groups (7.1% severe malaria)
Huerga H et al.^87^ Spain, 2002.	56	56 children	SSA	43 *P.falciparum,* 2 *P.malariae,* 2 *P.ovale,* 5 mixed (3 *P.falciparum* & *P.malariae*, 1 *P.m*alariae & *P.ovale*, 1 *P.falciparum* & *P.ovale*)	0
Huerga H et al.^88^ Spain, 2001	44	44 children	NS general data from different groups (98% SSA, 2% LA)	NS general data from different groups (78% *P.falciparum*, 12% *P.malariae*, 8% *P.ovale*, 2% *P.vivax*)	1
Matteelli A et al.^89^ Italy, 2001	22	22 Adults	Asia (China)	20 *P.falciparum,* 1 *P.ovale,* 1 *P.falciparum/P.ovale*	7

NS: not specified. SSA: Sub-Saharan Africa
